# Vaccine coverage, timeliness and delay estimated from regional and national cross-sectional surveys in Ethiopia, 2016

**DOI:** 10.11604/pamj.2021.39.205.22777

**Published:** 2021-07-19

**Authors:** Abram Luther Wagner, Yemesrach Abeje Tefera, Brenda Wilson Gillespie, Bradley Frederick Carlson, Matthew Lester Boulton

**Affiliations:** 1 Department of Epidemiology, School of Public Health, University of Michigan, Ann Arbor, Michigan, USA,; 2 Department of Public Health, St. Paul´s Hospital Millennium Medical College, Addis Ababa, Ethiopia,; 3 Department of Biostatistics, School of Public Health, University of Michigan, Ann Arbor, Michigan, USA,; 4 Department of Internal Medicine, Division of Infectious Disease, University of Michigan Medical School, Ann Arbor, Michigan, USA

**Keywords:** Ethiopia, vaccine coverage, vaccination timeliness, vaccine delay

## Abstract

**Introduction:**

measures of vaccine timing require data on vaccination dates, which may be unavailable. This study compares estimates of vaccine coverage and timing; and compares regression techniques that model these measures in the presence of incomplete data.

**Methods:**

this cross-sectional study used the 2016 Ethiopian Demographic and Health Survey (DHS), and a 2016 survey from Worabe, Ethiopia. Three measures of vaccine uptake were calculated: coverage (regardless of timing), timeliness (within 1 week of recommended administration), and delay (the number of days between the recommended and actual date of vaccination). Vaccine coverage and timeliness were modeled with logistic regressions. After excluding those without dates, vaccine delay was estimated using linear regression or survival analysis. Vaccine delay was also estimated using accelerated failure time (AFT) models.

**Results:**

the DHS survey included 3819 children aged 12-60 months and the Worabe survey included 484 children aged 12-23 months. In the Worabe survey, vaccine coverage for pentavalent vaccine dose 3 was 87.4%, with 8.6% receiving it within 1 week, and 71.7% within 4 weeks; the median delay was 19 days. Predictors of outcomes were similar in both the Worabe survey and Ethiopian DHS, with the largest numbers of significant associations seen in models with vaccine coverage or delays (with AFT models) as the outcomes.

**Conclusion:**

estimates of coverage may miss a substantial proportion of infants who have delayed vaccination. Accelerated failure time (AFT) models are useful to estimate vaccine delay because they include information from all respondents (those with full and partial data on vaccination dates) and are agnostic about an age limit for timely vaccination.

## Introduction

The World Health Organization (WHO) in 1974 launched the Expanded Program on Immunization (EPI), a set of recommendations for countries to publicly fund certain vaccines [1]. The goal of the EPI program was to provide immunization to every child for Bacillus Calmette-Guérin (BCG), oral polio vaccine (OPV), diphtheria-tetanus-pertussis (DTP) and measles. The EPI program has expanded to recommend hepatitis B vaccine, *Haemophilus influenza* type b (Hib) vaccine, rubella vaccine, pneumococcal conjugate (PCV), and rotavirus vaccine [1]. Individual countries may have national immunization schedules that deviate from the WHO EPI. However, Ethiopia has a relatively comprehensive schedule, including BCG, OPV, IPV, pentavalent (DTP, hepatitis B and Hib), PCV, and rotavirus [2]. Widespread use of vaccines has had a positive impact on the morbidity of vaccine preventable diseases. One study has shown a greater than 99% decrease in diphtheria, measles, polio, rubella and smallpox compared to disease rates before the EPI [3].

To assess vaccination program performance, various measures of vaccine uptake are used. The most common measure, vaccine coverage [4], is the proportion of individuals who reported at their interview to have received a vaccine among those age eligible. DTP3 (or pentavalent 3 for countries with this vaccine on their schedule) is commonly used in vaccination surveys to measure immunization system performance because it is one of the longest used vaccines and because it measures whether families are able to attend vaccination services across at least three visits. The pentavalent vaccine is used instead of DTP in Ethiopia and many other countries. Simply focusing on receipt of DTP3 misses out on timeliness of vaccination. For example, studies in the United States have shown that less than half of children had received all recommended vaccine doses on time [5], even though vaccination coverage is high (>90%) [5,6]. Delayed vaccination has been cited as a reason for over 10% of pertussis cases in the United States [3], and individuals under-immunized because they have not received all vaccine doses on time have been present in various measles [7] and pertussis [8] outbreaks. These findings have lead researchers to recommend the use of age appropriate indicators and vaccine timeliness in measuring the effectiveness of vaccine programs [9-13].

The best way to measure timeliness is unclear - at what point are doses considered timely versus delayed? Additionally, in many low resource settings, vaccination records (at home in the form of a vaccination card or at a clinic in the form of a paper or electronic record) may be unavailable [14], and thus an exact measurement of timeliness may not be available for every individual. Therefore, we undertook a study to show how timeliness can be measured-using both a small-scale survey and a nationally representative survey-within a low-income country. Data from two sources (a 2016 cross-sectional survey of mothers in the town of Worabe, Ethiopia, and the 2016 country-wide Ethiopia Demographic and Health Survey (DHS)) were used to (1) compare estimates of vaccine coverage, timeliness, and delay; and (2) compare different regression techniques that model measures of vaccine uptake.

## Methods

**Worabe study population:** this cross-sectional study took place during July and August 2016 in Worabe, a town 100 miles south of the capital Addis Ababa in in the Southern Nations, Nationalities, and Peoples´ Region. Most individuals in the town come from the Silte ethnic group, and the town comprises 27,852 residents according to 2007 Ethiopian census. The sampling criteria has been described previously [15]. Briefly, interviewers enumerated households within *kebele*, a neighborhood-level unit in Ethiopia, selected a random starting point, and then systematically selected households thereafter. Households were included if they had a child 12-23 months of age. Only the mother of the enrolled child was interviewed. The sample size calculation was based off another aim [15], i.e. to have precise confidence intervals around an outcome of full vaccination coverage, which we supposed to be 80%. With a marge of error of 5%, a 10% non-response rate, and a design effect of 2, we need a sample size of 541.

**Demographic and health survey study population:** the 2016 DHS survey collected data from 15,683 women ages 15-49 and 7,814 children ages 12-60 months from each of the 11 regions in Ethiopia. The 2016 DHS sample was selected using a stratified, two-stage cluster design. Administratively, regions in Ethiopia are divided into zones, and zones are split into administrative units called *wereda*. Each *wereda* is further subdivided into the lowest administrative unit, called kebele and each kebele was subdivided into census enumeration areas (EAs). The sample included 645 EAs (202 in urban areas and 443 in rural areas). Households comprised the second stage of sampling. A complete listing of households was carried out in each of the 645 selected EAs from January 2016 through June 2016. DHS anonymizes their survey locations and we were unable to verify if the DHS survey had also been conducted in Worabe. DHS data are publicly available [16]. The DHS sample size is designed to generate nationally and regionally representative statistics [17].

**Outcome definitions:** within this paper, “vaccine uptake” is a generic word encompassing all other measures of vaccination status. “Vaccination coverage” refers to receipt of a vaccine, regardless of its timing. Timeliness is a measure of vaccination occurring within a specific age range; within this paper, it is calculated both within 1 week and within 4 weeks of its recommended administration. “Vaccination delay” refers to the time elapsed between the recommended date of vaccination, and the actual date of vaccine administration. Information about vaccines was obtained from a vaccination card (with dates available) or from maternal recall (with no dates available). In the Worabe study, vaccination cards with dates were almost universally available. In the DHS, a large minority of participants did not have a usable vaccination card.

**Statistical analysis:** overall the statistical methods in this study were selected to evaluate how methods and construction of variables can influence interpretation of results, even with a singular aim. Vaccine uptake was estimated using proportions and standard errors. For the Worabe group, the distributions of vaccine delay in days, available from the vaccination cards, were estimated using medians and interquartile ranges (IQR). Several multivariable regression models were run in both the Worabe and DHS datasets using data on pentavalent dose 3. For the outcomes of vaccine coverage and timeliness of administration within 1 week and within 4 weeks of recommended vaccination, we used logistic regression models. For the outcome of vaccine delay, a Cox regression model was used. For those with vaccination cards, the date of vaccination minus the date of birth was the time of event, and those who had no vaccination recorded were right censored at their age of the interview. A method included for completeness was a linear regression of vaccination by age in days between recommended and actual date of vaccination. The timeliness models, linear regression, and Cox proportional hazards model only used data from individuals who had a vaccination card with dates. If many individuals do not have a card, then this method is subject to bias from exclusions. A method to account for both left-censored data (the mother reports the child was vaccinated but does not have vaccination card with date) and right-censored data (the individual not receiving a vaccine by the time of interview) was possible using accelerated failure time (AFT) models. The AFT models used a Weibull distribution, which had better fit than AFT models based on the lognormal and gamma distributions (results not shown). The AFT model coefficients were exponentiated to calculate acceleration factors (AF), which were interpreted as the expected time to vaccination in one category relative to the referent group.

Explanatory variables were selected into the models *a priori*. For the Worabe dataset, four explanatory variables were entered into every model: maternal occupation, owns farm land, number of antenatal care visits, and distance to vaccination site. These four variables were based on the significant predictors of vaccination coverage from a previously published study [15]. Within the Ethiopia DHS dataset-occupation, number of antenatal care visits, home delivery, and ethnic group-were chosen based on overlap with variables in the Worabe dataset and significant variables from a previously published study [18]. Significance was assessed at a level of α=0.05. Data were analyzed in SAS software version 9.4 (SAS Institute, Inc., Cary, NC). Documentation of code used in this study and how to implement AFT models is publicly available [19].

**Ethical approval:** the Institutional Review Board of St Paul´s Hospital Millennium Medical College in Ethiopia reviewed and approved the Worabe study proposal (approval #P.M.23/17). Permission to undertake the study was granted by the Worabe Health Bureau. Verbal informed consent from mothers of the young children was obtained prior to enrolling participants into the study. No ethical approval was sought for analyzing the Ethiopia DHS dataset because it is freely and publicly available and contains no personally identifiable information.

## Results

**Worabe study:** out of the 540 mothers of children aged 12-23 months that were approached to be enrolled in the study, 484 (90%) agreed. Half (241, 50%) of the children were female. About half (51%) of mothers were house wives, the rest were students (1%) or had jobs outside of the home. About one-third (162, 33%) owned farm land. Many mothers had either ≥4 antenatal care (ANC) visits (208, 43%), or 2-3 visits (146, 30%); 27% (130) had had 0-1 visits. The distance between the individual´s house and the vaccination site was relatively evenly split between taking <30 minutes (157, 32%), 30 to 59 minutes (181, 37%), or ≥60 minutes (146, 30%).

Table 1 shows different measures of vaccine uptake for each vaccine dose on the Ethiopia EPI. In general, overall coverage, as measured among those with a vaccination card, was high: above 95% for birth doses (BCG or OPV0), and dose 1 of vaccine series started at 6 weeks (OPV, pentavalent, PCV, or rotavirus), but declining to 87% for dose 3 of these series, and to 83.6% for measles vaccine, which is given at 9 months. For the vaccine series started at 6 weeks, less than half were administered within 1 week of the recommended date, and there were greater delays for subsequent doses. For example, 95.8% of children received pentavalent dose 1, according to the vaccination card. Only 44.4% received it within 1 week, although 94.7% did within 4 weeks. By dose 3 of this series, coverage was 87.4%, but only 8.6% had received it within 1 week, and 71.7% had received it within 4 weeks of the recommended date. The median number of days between recommended vaccination date and actual vaccination date increased from 7 (IQR: 5, 11) to 19 (IQR: 12, 28) between dose 1 and dose 3 of this series.

**Table 1:** measures of vaccine uptake among children 12-23 months of age in Worabe, Ethiopia

	Coverage proportion			Timeliness proportion		Delay: median (IQR)
	Card	Mother´s recall	Card + recall	Within 1 week	Within 4 weeks	After EPI recommended schedule
**BCG**	97.1	95.1	96.0	48.5	58.7	7 (0, 49)
**OPV0**	95.8	97.1	96.1	71.9	87.7	3 (0, 7)
**OPV1**	95.8	96.7	95.9	44.7	94.7	7 (5, 11)
**OPV2**	94.5	91.8	92.8	16.1	92.9	12 (8, 16)
**OPV3**	87.8	69.3	78.1	9.0	70.5	18 (12, 29)
**Pentavalent 1**	95.8	96.3	95.4	44.4	94.7	7 (5, 11)
**Pentavalent 2**	94.5	95.1	94.2	16.9	92.8	12 (8, 16.5)
**Pentavalent 3**	87.4	82.3	84.3	8.6	71.7	19 (12, 28)
**PCV1**	95.8	96.7	95.6	44.6	94.6	7 (5, 11)
**PCV2**	94.5	95.5	94.4	17.5	92.2	12 (8, 17)
**PCV3**	87.0	82.7	84.3	9.2	71.7	19 (12, 28)
**Rotavirus 1**	95.8	84.7	89.5	44.3	94.0	7 (5, 11)
**Rotavirus 2**	95.4	81.4	87.6	17.4	91.6	12 (8, 17)
**Measles**	83.6	80.9	82.2	14.7	30.6	45.5 (19, 72)

BCG: bacillus calmette-guérin; OPV: oral poliovirus vaccine; PCV: pneumococcal conjugate vaccine; IQR: interquartile ranges; EPI: expanded program on immunization

Multivariable regression models of pentavalent dose 3 are shown in Table 2. Compared to children whose mothers had ≥4 ANC visits, those with 0-1 ANC visits had 0.05 times the odds of pentavalent dose 3 coverage (95% CI: 0.01, 0.25). According to the AFT model, the expected time to pentavalent dose 3 vaccination was 2.67 times greater for those with 0-1 ANC visits (95% CI: 1.92, 3.72) and 1.43 times greater for those with 2-3 ANC visits (95% CI: 1.08, 1.90), compared to children whose mothers had ≥4 visits. For this model, 151 (31%) of values were noncensored, 132 (27%) were right censored, and 200 (41%) were left censored.

**Table 2:** predictors of pentavalent dose 3 uptake, across different models and measures of uptake, among children 12-23 months of age in Worabe, Ethiopia, 2016

	Coverage	Timeliness (in 1 week)	Timeliness (in 4 weeks)	Delay in days (only including vaccination dates)	Delay	Delay
	Logistic regression OR (95% CI)	Logistic regression OR (95% CI)	Logistic regression OR (95% CI)	Linear regression β (95% CI)	Cox proportional hazards HR (95% CI)	Accelerated Failure time model AF (95% CI)
**Sample size**	484	152	152	151	151	483
**Occupation**						
House wife	ref	ref	ref	ref	ref	ref
Other	0.79 (0.46, 1.38)	1.55 (0.48, 5.05)	1.15 (0.53, 2.50)	-3.26 (-8.40, 1.89)	1.16 (0.82, 1.62)	0.87 (0.68, 1.12)
**Farm Land**						
No	ref	ref	ref	ref	ref	ref
Yes	1.14 (0.62, 2.10)	0.61 (0.15, 2.47)	0.63 (0.29, 1.40)	3.62 (-1.82, 9.05)	0.79 (0.53, 1.14)	1.30 (1.00, 1.69)
**Number of antenatal care visits**						
0-1	0.06 (0.03, 0.14)**	0.75 (0.14, 4.22)	0.41 (0.16, 1.03)	2.78 (-3.77, 9.33)	0.79 (0.51, 1.21)	2.67 (1.92, 3.72)**
2-3	0.25 (0.11, 0.60)*	1.44 (0.40, 5.21)	1.13 (0.46, 2.75)	-1.74 (-7.42, 3.95)	1.10 (0.75, 1.61)	1.43 (1.08, 1.90)*
≥4	ref	ref	ref	ref	ref	ref
**Distance to vaccination site**						
<30 minutes	0.73 (0.38, 1.40)	2.73 (0.64, 11.70)	1.27 (0.53, 3.01)	-3.89 (-9.79, 2.01)	1.23 (0.83, 1.81)	1.23 (0.92, 1.64)
30 to 59 minutes	ref	ref	ref	ref	ref	ref
≥60 minutes	0.81 (0.41, 1.59)	2.19 (0.45, 10.81)	1.99 (0.77, 5.11)	-6.38 (-12.52, -0.24)*	1.52 (0.98, 2.34)	0.79 (0.59, 1.07)

* P<0.05, **P<0.0001; Note that the directionality is opposite for Cox hazard ratios and accelerated failure time coefficients. AF: acceleration factor; HR: hazards ratio; OR: odds ratio

Although not all parameter estimates were significant in all models, the directionality was generally consistent. For example, owning farm land was not significantly associated with any vaccination uptake outcome. However, the odds were estimated to be <1 (e.g. lower) among those with farm land compared to those without, for measures of vaccine coverage and both measures of vaccine timeliness. The amount of delay was correspondingly positive in the linear regression model. Across the different models, 2 parameters were significant with the outcome of vaccination coverage, 0 were significant for both timeliness outcomes, 1 was significant for vaccination delay (using the Cox model with age at vaccination or right censoring for not yet vaccinated), and two were significant for vaccination delay when using the AFT model to include both right- and left-censoring.

Demographic and health survey study: of the 7,814 mothers in the DHS sample, 3,819 had children 12-60 months of age who had vaccination information. Of these children, 49% (1,886) were females. Ethnically, 24% (932) identified as Oromo, 15% (584) Amhara, 11% (334) Tigray, 13% (507) Somali, 9% (349) Afar and 26% (1007) as other ethnicities. When asked about occupation, 58% (2,213) of the children´s mothers reported that they did not work. The majority (62%, 2,365) of these children´s mothers reported giving birth at home; only 31% (1,178) of mothers had ≥4 antenatal care (ANC) visits, 21% (790) had 2-3 visits (790, 21%); and 48% (1,843) had 0-1 visits.

Table 3 shows predictors of pentavalent dose 3 uptake according to the 2016 Ethiopian DHS. Having fewer antenatal care visits was associated with worse vaccine pentavalent dose 3 uptake outcomes; for instance, compared to children whose mothers had ≥4 ANC visits, children whose mothers had 0-1 ANC visits had only 0.27 times the odds of receiving the vaccine regardless of time (95% CI: 0.23, 0.33), had 0.67 times the odds of receiving the vaccine within 4 weeks (95% CI: 0.52, 0.87), had vaccination delayed 25.81 days (95% CI: 12.74, 38.88), and their rate of vaccination was only 0.75 times as high (95% CI: 0.66, 0.85). In the AFT model, the expected time to pentavalent dose 3 administration was 0.83 times as high among children whose mothers were working outside of the home compared to those whose mothers did not (95% CI: 0.75, 0.92), and this delay was significantly greater for those whose mothers had 0-1 or 2-3 ANC visits compared to those whose mothers had had ≥4 ANC visits. Children whose mothers delivered at home and certain ethnic groups also had significantly greater delay. In this model, 1663 (43%) of values were non-censored, 1883 (49%) were right censored, and 303 (8%) were left censored. Overall, 9 parameters were significant when using the outcome of vaccine coverage, 3 were when using vaccine timeliness at 1 week, 4 were when using vaccine timeliness at 4 weeks, 5 were with the outcome of delay in days, 5 were when using a Cox proportional hazards model, and 9 were using the AFT model.

**Table 3:** predictors of pentavalent dose 3 uptake in the 2016 Ethiopian demographic and health survey

	Coverage	Timeliness (in 1 week)	Timeliness (in 4 weeks)	Delay in days (only including vaccination dates)	Delay	Delay
	Logistic regression OR (95% CI)	Logistic regression OR (95% CI)	Logistic regression OR (95% CI)	Linear regression β (95% CI)	Cox proportional hazards HR (95% CI)**	Accelerated Failure time model AF (95% CI)**
**Sample size**	3813	1669	1669	1669	1663	3849
**Occupation**						
Not working	ref	ref	ref	ref	ref	ref
Working	1.38 (1.18, 1.61)**	1.21 (0.95, 1.53)	1.09 (0.89, 1.34)	-0.62 (-10.89, 9.66)	1.03 (0.93, 1.14)	0.83 (0.75, 0.92)*
**Number of antenatal care visits**						
0-1	0.27 (0.23, 0.33)**	0.79 (0.58, 1.07)	0.67 (0.52, 0.87)*	25.81 (12.74, 38.88)*	0.75 (0.66, 0.85)**	2.80 (2.45, 3.19)**
2-3	0.68 (0.55, 0.85)*	0.78 (0.58, 1.04)	0.72 (0.56, 0.92)*	15.99 (3.51, 28.47)*	0.80 (0.71, 0.90)*	1.33 (1.17, 1.50)**
≥4	ref	ref	ref	ref	ref	ref
unknown	0.30 (0.06, 1.52)	1.05 (0.11, 10.32)	0.20 (0.02, 1.93)	43.36 (-58.40, 145.12)	0.56 (0.17, 1.30)	1.93 (0.67, 5.57)
**Delivery**						
At home	0.43 (0.36, 0.50)**	0.59 (0.45, 0.76)**	0.45 (0.36, 0.56)**	42.27 (31.39, 53.14)**	0.64 (0.57, 0.71)**	1.98 (1.78, 2.21)**
Not at home	ref	ref	ref	ref	ref	ref
**Ethnic group**						
Afar	0.11 (0.07, 0.18)**	0.91 (0.29, 2.80)	0.97 (0.39, 2.44)	38.73 (-6.02, 83.48)	0.78 (0.49, 1.17)	8.01 (5.05, 12.70)**
Amhara	1.58 (1.25, 2.01)*	0.97 (0.68, 1.36)	1.41 (1.04, 1.91)*	-16.86 (-32.09, -1.63)*	1.32 (1.14, 1.53)*	0.68 (0.59, 0.79)**
Oromo	ref	ref	ref	ref	ref	ref
Somali	0.65 (0.51, 0.84)*	1.96 (1.21, 3.18)*	1.08 (0.68, 1.70)	-7.30 (-30.10, 15.50)	1.09 (0.87, 1.36)	1.43 (1.16, 1.76)*
Tigray	2.55 (1.90, 3.41)**	0.59 (0.40, 0.86)*	1.11 (0.81, 1.51)	-17.82 (-33.48, -2.17)*	1.23 (1.05, 1.43)*	0.56 (0.48, 0.66)**
Other	1.50 (1.23, 1.82)**	0.92 (0.67, 1.27)	1.04 (0.79, 1.38)	1.60 (-12.39, 15.58)	1.00 (0.88, 1.15)	0.74 (0.65, 0.85)**

* P<0.05, **P<0.0001; **Note that Cox hazard ratios (higher is better) and accelerated failure time coefficients (higher is worse) are scaled in opposite directions. AF acceleration factor; HR: hazards ratio; OR: odds ratio

## Discussion

Compared to traditional vaccination coverage estimates, measures of timeliness and delay more accurately reflect the timing of the vaccination event [12], and therefore may be a better descriptor of population-level immunity and health care access [20]. In its comparison of a variety of vaccination uptake measures, this study found broad concordance in results (e.g. having more ANC visits was associated with an infant having greater uptake of vaccine, better timeliness and less delay). However, by including information from those both with and without specific timing data (i.e. vaccination cards), measures of delay using AFT models may allow researchers to study a wider range of variables and have greater statistical power to detect disparities.

Previous interaction with health care providers was an important predictor of vaccination timeliness in both the national and Worabe samples. Previous studies have shown that access to health care workers presents opportunities for education of mothers regarding vaccine administration [21]. This was demonstrated in our findings in that mothers who delivered their child at home and those with ANC visits had a significantly greater delay in vaccination. A study in India has found that a lack of antenatal care was highly predictive of non-vaccination [21]. In another study from India, both antenatal care and location of delivery had a substantially larger effect on non-vaccination than on under-vaccination. Children born in an institutional setting were far more likely to be vaccinated than those who were born outside of an institution [22]. Birth setting, particularly public institutional birth compared with home-delivery, has been shown to be associated with increased odds of complete vaccination in Kenya and elsewhere [23]. An analysis of vaccination data from Kenya has provided some evidence for the mechanisms behind greater exposure to health care within institutions and vaccination timeliness. Kenyan children who had birth in an institutional setting were more likely to have co-administered vaccine doses at birth and at 6 months, and co-administration of these doses was associated with more timely vaccinations later in the infant´s first year of life [24]. Vaccine hesitancy is present within the Ethiopian population [25], and continued conversations with doctors and nurses could decrease this. In summary, linking a pregnant woman and an infant to health systems early on can lead to a sustained increase in the timeliness of the receipt of health care services, including vaccination.

**Comparison of vaccine uptake measures:** within the Worabe study, we found overall high coverage of vaccination but a substantial proportion of infants with delayed or untimely vaccinations. Significance of results is a function of the actual effect of an explanatory variable on an outcome, as well as the sample size of the dataset. Thus, any analysis that is able to incorporate more information from more variables (e.g. the vaccination coverage and delay in days with AFT models, which include data from all observations, vs. timeliness or delay measures which only include data from those observations with vaccination cards) will have greater numbers of significant variables. However, compared to a measure of delay, vaccination coverage ignores the question of when people were vaccinated. In the presence of substantial delay, coverage will of course be greater than timeliness, and coverage will also be greater as the average age in the study population increases.

We also believe that it is important to consider, as previous manuscripts suggest [24], that children with and without vaccination cards may be sociodemographically different, and may have different vaccination outcomes. By excluding children without vaccination cards, the study population may be biased. However, it is also important to note that recall of vaccination information may be less valid than information obtained directly from clinics or from vaccination cards [26]. Overall, this study shows the importance of incorporating as much information in statistical models as possible. For vaccination, information on timing could be incorporated in the construction of the outcome (vaccination timeliness or vaccination delay) and in the specification of multivariable models (through AFT models, for instance).

**Strengths and limitations:** this study used two different datasets from Ethiopia in 2016 to demonstrate how calculating different measures of vaccine uptake could be applied to both small scale and nationwide surveys. However, both surveys were cross-sectional, and our interpretation of the substantive findings are limited with the lack of longitudinal information; however, most explanatory variables selected-number of antenatal care visits, ethnic group, possession of farm land-would not have been influenced by the vaccination status of the child. We relied on respondent self-report, and there could be measurement bias in the explanatory variables, although we are unsure what direction this would be in and how it would impact the results.

## Conclusion

Many measures of vaccine uptake are available, but the trend in recent years has been to emphasize measures of vaccine timing - which include some information about vaccination dates. However, in many low resource settings, exact vaccination dates may be unavailable for analysis. In both the Worabe survey and Ethiopian DHS, the regression models showed similar trends in the directionality of the association between explanatory variables and the outcome (including measures of vaccine coverage, vaccine timeliness, and vaccine delays). The largest number of significant associations were seen when using vaccine coverage or delays (using an AFT model) as the outcomes. Using an AFT model is a reasonable approach which balances including information from all respondents (those with and without data on vaccination dates) and which is agnostic about what age demarcates a timely vs untimely vaccination.

### What is known about this topic

- Some measures of vaccine uptake do not consider timing of the vaccine;
- Measures of vaccine timing require vaccination dates, which may be unavailable for a large fraction of the study population.

### What this study adds

- Use of accelerated failure time models incorporates vaccine timing information, even among those without vaccination cards;
- Accelerated failure time models can be feasibly used for large and small-scale studies.
